# Adolescents’ Experience of Pain: A Focus Group Study in Sweden

**DOI:** 10.1177/10598405231212907

**Published:** 2023-11-13

**Authors:** Camilla Perming, Eva-Lena Einberg, Sarah Bekaert, Pernilla Garmy

**Affiliations:** 1 Department of Nursing and Health Sciences, Faculty of Health Sciences, 4342 Kristianstad University, Kristianstad, Sweden; 2 Oxford Institute of Applied Health Research, 98464 Faculty of Health & Life Sciences, Oxford Brookes University, Oxford, UK; 3 Department of Health Sciences, Faculty of Medicine, 5193 Lund University, Lund, Sweden

**Keywords:** high school, pain, qualitative research, mental health

## Abstract

The purpose of this study was to describe experiences of pain among students at upper secondary schools (adolescents aged 16–19 years) in Sweden. Focus group (n = 9) interviews with 41 adolescents were conducted in southern Sweden in 2021. The interviews were analyzed by qualitative content analysis, which identified four categories encompassing the theme *how pain expressed itself*: (1) a warning bell—the body's way of saying that something is wrong; (2) school- and work-related pain; (3) interpersonal physical and emotional pain; and (4) heartache—a sign of humanity; and four categories describing the theme *ways of dealing with pain*: (1) turn your attention elsewhere; (2) “bite the bullet” and endure the pain; and (3) use painkillers or complementary treatments; and (4) ask for help from others. Findings are linked to the crucial role of school nurses in supporting students who experience pain.

## Background

Pain among adolescents is common and there is evidence suggesting that they experience pain more frequently in recent compared to previous years (Gobina et al., 2019). The Health Behavior in School-aged Children (HBSC) study of over 200,000 children and adolescents aged 11–15 from 42 countries, showed that 44% of adolescents reported weekly pain during the last six months (Gobina et al., 2019). The health behavior study with Swedish School-aged Children (2021/2022) shows that the proportion of 15-year-old girls who have had headaches more than once a week in the last 6 months had increased from 30% in the previous survey, to 45%. Similar patterns can be seen for stomach pain. Among 15-year-old boys in Sweden, the proportion with headaches has remained stable over the same period, at around 10%. However, in the past 30 years (1985/1986 to 2021/2022), there has been an increase in headaches, stomach, and back pain reported by among 15-year-old adolescents, especially among girls (Public Health Agency of Sweden, 2023).

Pain is rarely an isolated experience and is frequently the consequence of physical and/or social challenges. Studies of adolescents have explored specific pain-related associations. For example, a cross-sectional survey with adolescents in Iceland examined the use of analgesics and the association with being a victim of bullying among adolescents (Garmy et al., 2019). In addition, chronic or recurrent pain can precipitate further issues, such as stress and depression. Borgman et al.'s (2020) cross-sectional survey with adolescents in Sweden describes the prevalence of pain (headache, abdominal pain, and back pain) and its relation to depressive symptoms. The consequences of recurrent pain can also be cumulative through a negative impact on academic performance (Ragnarsson et al., 2020) and is associated with long-term risk for socioeconomic and social disparities (Murray et al., 2020).

Such studies focusing on adolescent pain have mainly been quantitative. Adolescents are at a unique physical, cognitive, and psychosocial developmental stage, and therefore there is a need to attend to and explore their specific experience of pain across these stages of the lifespan. There is a need to listen to the voices of adolescents and to explore how they experience and manage pain. The International Association for the Study of Pain (IASP) defines pain as “an unpleasant sensory and emotional experience associated with, or resembling that associated with actual or potential tissue damage” (Raja et al., 2020). The biopsychosocial model of pain specifies that pain consists of nociception, perception, affect, and behavioral and environmental factors (Nicholas, 2022). Pain is, however, also a subjective experience. McCaffery and Beebe (1989) stated that pain is whatever the experiencing person says it is and exists whenever the experiencing person says it does. In addition, there is a tendency to place pain in the medical model, referring to acute and chronic pain—however, here we seek to explore pain in a non-clinical space, allowing for the experience of pain beyond the pathophysiology and as part of what it means to be human. The focus group study reported here is unique in that it gave adolescents a qualitative space to describe and discuss their experience of pain.

The school nurse plays a vital role in supporting children and adolescents who are experiencing pain. The Swedish Education Act (2010) outlines the role of the school nurse in health education and promotion. The school nurse has a knowledge base in public health and child development and is able to form supportive relationships with children and adolescents. Their role in pain management is twofold: to exclude a medical cause for the pain, and to equip the young person to manage their pain (Golsäter et al., 2012). Wakefield et al. (2018) found that adolescents perceive stigma surrounding chronic pain, which is a barrier to help-seeking. Some report feeling that complaints of pain will not be believed or taken seriously, and they fear being unable to obtain the support they need. The school nurse is also an accessible health professional for children and adolescents. In Sweden, children can independently access the school nurse at school, school staff or parents may raise a concern for a child, or an issue may be identified at the regular assessment, which is part of the national health monitoring program. Consequently, the school nurse is uniquely placed to support adolescents in managing pain.

## Aim

The aim of the current study was to describe experiences of pain among adolescents aged 16–19 years in upper secondary schools to inform school nursing practice.

## Methods

This is an inductive qualitative focus group study using the consolidated criteria for reporting qualitative research (COREQ) guidelines (Tong et al., 2007). The study is part of a larger study that explored pain and stress. The domains of stress and pain were explored separately within each focus group. A convenience sample within several secondary schools was recruited. Due to the busy school schedule, there was a need to maximize the time available with the young people. Therefore stress and pain were explored as separate, yet interrelated concepts. Results focusing on adolescent's experience of stress are published elsewhere (Perming et al., 2022). The study was approved by the Swedish Ethical Review Authority (2018/842).

## Data Collection

The research team approached principals at six upper secondary schools in southern Sweden with information about the study. Two schools with approximately 400 students each accepted the invitation to participate. Written information about the study was provided to the students and their guardians. Forty-one adolescents aged 16–19 (22% males/78% females) gave written informed consent and were interviewed in focus groups (n = 9) during 2021. The mean age was 17.5 (range 16–19). The interviews were audio-recorded and transcribed verbatim by the interviewers.

All focus groups were held at school during the school day. The mean time was 45 min (range 30–90 min). Four focus groups consisted of females only, and five consisted of females and males. The median size of the focus groups was five participants (range 3–6). The first author took part in all interviews together with a Master's level researcher. They were both female, with experience as registered nurses. The interviewers were supervised by the second and fourth authors, who are pediatric nurses with doctoral degrees proficient in focus group interviews with children and adolescents. The researchers and the respondents had no relationship before study commencement.

The interview guide included open questions to invite description and discussion (see Appendix). The interview guide was developed for the age group 10–19, and has been validated through previous use among 10–12-year-old children (Persson et al., 2021). Using the same guide with teenagers offers the opportunity to examine developmental differences between the age groups in their understanding and description of pain and tailor school nurse support accordingly. The interview schedule was piloted with one focus group in this older age group, adding further rigor to the research process. No changes to the schedule or process were indicated through the pilot.

## Data Analysis

Hsieh and Shannon's (2005) conventional qualitative content analysis was used to analyze the data. Each transcript was then reviewed by the two interviewers, and discussed to ensure that the content was perceived similarly and that nothing was missed. In the next step, the first and last authors individually read each transcript carefully, highlighting text that appeared to describe experience of pain. Collaboratively, text-related passages were grouped together under headings or developing codes. After open coding of four transcripts, preliminary codes were decided. The researchers then individually coded the remaining transcripts using these codes and adding new codes when they encountered data that did not fit into an existing code. Once all transcripts had been coded, they collectively examined all data within a particular code reaching agreement through discussion. Some codes were combined during this process, whereas others were split into two. In the next step, the researchers examined the final codes to organize them into subcategories and categories, see Table 1. Finally, all four authors met and discussed the analysis until consensus was reached.

**Table 1.** Description of Adolescents’ Experiences of Pain, Themes and Categories.

Themes	Categories
How pain expressed itself	Warning bell—the body's way of saying that something is wrong
	School- and work-related pain
	Interpersonal physical and emotional pain
	Heartache—a sign of humanity
Ways of dealing with pain	Turn your attention elsewhere
	To “bite the bullet” and endure the pain
	Use of painkillers and complementary treatments
	Ask for help from others

## Results

The analysis identified two major themes. Four categories describing the theme *how pain expressed itself:* (1) a warning bell—the body's way of saying that something is wrong; (2) school- and work-related pain; (3) interpersonal physical and emotional pain; and (4) heartache—a sign of humanity; and four categories describing the theme *ways of dealing with pain:* (1) turn your attention elsewhere; (2) “bite the bullet” and endure the pain; (3) use painkillers or complementary treatments; and (4) ask for help from others, see Table 1.

### How Pain Expressed Itself

The adolescents moved between descriptions of physical and emotional pain: from the physical pain of a sports injury or muscle aches, to the stress of overwork, to the emotional pain of romantic relationships or the loss of a loved one. There was an overarching sense that pain, while unpleasant, could be a positive entity. Experiencing pain signaled that something was wrong, whether that be needing to move position while studying, or a negative relationship. Pain was also noted to transform into knowledge, and, in the case of grief, positive memories in the longer term.

### A Warning Bell

The adolescents classified pain as a negative experience, but elaborated that pain was a warning bell—a way for the body to signal that something is wrong. They believed that pain was necessary and beneficial as an alarm, although the pain itself was perceived as unpleasant. For example, experiencing headaches, back pain, or stomach pain as a result of stress related to school or activities outside of school, such as sport-related events. Others reported that they could feel pain and heart palpitations in stressful situations, which most participants identified as a warning bell for stress. For example, one young person stated: “If you feel stressed, you can get a headache. It is the body's way of telling it that now it is a little too much” (Girl, Focus group 1). This highlighted the value of pain as a signal that her body had reached its limit. Another said: “The pain can be a good warning of something worse, so you can get a hold of it in time, so that you notice that there is something wrong too” (Girl, Focus group 4), emphasizing that pain can serve as a useful warning sign of a more severe problem, allowing individuals to act before it worsens.

### School- and Work-Related Pain

There was a close relationship described between stress and pain. The adolescents discussed the physical pain caused by stress and pressure associated with school and/or work. They mention feeling pain in their back and neck from sitting for long periods in uncomfortable positions.

The adolescents discussed how stress at school could lead to pain such as headaches and stomachaches. This pain was perceived as negative. If they felt like their schoolwork was overwhelming, the associated pain decreased their motivation to complete schoolwork and affected their academic performance: “When I thought a lot about school, I feel more pain about the stress and the pressure… during school time you aren’t in control. It hurts so much, so you have to concentrate during school, but you can’t” (Girl, Focus group 3). The adolescents gave examples from when they had a lot of work to do, but their resultant headache forced them to lie down to rest or sleep, thereby protracting the completion of their workload, generating additional stress. Some reflected that it was related to physical positioning. Some chose to endure such pain:

Yes, I have a lot of pain in my back and everything. I often sit in the lessons and crack my back because I’ve been standing bent over things; for, like, several hours I sit like this and, like, it's, like, the whole back and neck is cracking like hell. Like, it's still worse sometimes. (Boy, Focus group 7)

### Interpersonal Physical and Emotional Pain

The adolescents reported experiencing pain in relation to interpersonal relationships. This pain was reported to be both physical and emotional.

One adolescent experienced physical violence from others. He expressed that the anger and adrenaline took away the pain in the moment. After calming down emotionally, the pain was noticeable once again and manifested as physical pain in the bodily regions where he had been hit:

Yeah, when it comes to physical pain, when I get angry, then I don’t feel physical pain. Some guys tried to rob me once, those, those bastards knocked me 10–15 times on the ground. I stood up every time, spat blood on the ground, and said “Go to hell. It's my stuff.” I get so damn angry I don’t feel pain. They had kicked the hell out of my whole face and I just, no… After that I couldn’t eat for 2 weeks. (Boy, Focus group 7)

Another young person had experienced not feeling any pain when “taking a fall” for someone else. In making sure the other person was alright, the young person's physical pain became easier to endure:

Or, like, once in the internship, there was a child who was about to fall down a flight of stairs, and then I saved the child; I grabbed the child and then I fell, and I think that the pain should be on me and not the child. It was like I saved it… I saw the child was wobbling, so I thought I’ll get up, and I lifted the child up so it wouldn’t fall, then I was about to lose my balance, and so he landed on me, so he didn’t get hurt. People asked if I was okay like that and [I replied] “yes I just got a bit of a pain in my back but it's worth it.” (Girl, Focus group 5)

Emotional pain tended to be felt in relationships with close family members or friends. One young woman described the pain of interpersonal emotional conflict with peers:

If you are mean to someone who is not mean to you, then you can feel a lot of guilt for it and feel bad about it. (Girl, Focus group 6)

Such pain appeared more transient and was reported to dissipate once the conflict was resolved or once they forgave or were forgiven for a given transgression.

Family conflicts were described as painful situations, for example, if you have had a conflict with your family, it might not work right away (Boy, focus group 6). Some family-related difficulties hinted at deep ongoing challenges. For example, one adolescent stated, “my childhood hurts” (Girl, Focus group 8), which, for reasons of confidentiality could not be explored in a focus group scenario.

### Heartache—A Sign of Humanity

The adolescents also shared experiences of emotional pain that went beyond day-to-day relationships. Several adolescents reported experiencing emotional pain from a past romantic relationship (e.g., being cheated on, breaking up with someone they loved): “I think about if you’ve been hurt and if you’ve been devastated; you’ve had one relationship, but then ended, it's also a pain after all. Yes, so it is both psychological and physical, I think” (Girl, Focus group 5). This type of pain was described as a negative experience that was hard to mend, however, it was also described as making them stronger. This type of pain made them feel more resilient and wiser, from having learned a lesson that informed their behaviors in the future to avoid re-experiencing emotional pain: I think that mental pain can also strengthen you. You have everything you have been through with you, so maybe in the future you still feel somehow happy about it … that you have still learned something (Girl, Focus group 1).

The grief through death of a loved one was also discussed. The young people reported that, although losing a loved one engendered overwhelming pain, this pain could be transformed into a positive experience. One young woman explained that although grief was hard, it was a way of expressing the pain of loss:

One way can be some kind of pain in the heart. That you may have been devastated by something, a thing that you loved for a very long time; for example, a family member or something. It can also be a way of showing that pain. (Girl, Focus group 1)

Although, another dimension of the pain of such loss was that feeling such pain made them feel “alive,” that such grief is normal human emotion and a sign of humanity. They reported that, regardless of the emotional pain they felt, mourning a loved one was a transformative necessity in life: “There are many people who have lost something that they say it hurts a lot at the beginning, but then at the end it becomes a good pain like this, because it becomes memories instead” (Girl, Focus group 9).

### Ways of Dealing with Pain

The adolescents described a range of ways they dealt with pain. These included both positive strategies such as distraction and putting things into perspective, and negative strategies such as self-harming behaviors. Practical aspects were also discussed: seeking help from family, friends, and professionals; facing the pain head-on; and traditional treatments such as medication, as well as a range of alternative treatments.

### Turn Your Attention Elsewhere

The adolescents had various ways of handling pain. Many adolescents took a pragmatic approach to dealing with pain, for example, distraction and taking a philosophical approach, that is, thinking about the bigger picture. However, some spoke of self-harming behaviors. Although self-harm provided temporary relief, they knew that it was not beneficial in the long run.

Doing something you enjoy could help manage the pain, for example:

Maybe you watch a movie or you can think of something else … I usually talk to my mom and sometimes she might buy chocolate, it can make you very happy again… small things can make a very big difference. (Girl, Focus group 3)

Performing an act of kindness for someone else could also help manage the pain: “Make someone else happy and you will be happier yourself” (Boy, Focus group 9). Distractions included trying to think of something else, or putting things into perspective, and thinking that it could have been worse. Trying to laugh at the pain could also help:

Instead of being negative and “just how much it hurts” maybe you can laugh about it instead. … Try to think that worse things can happen, try to do something good instead. (Boy, Focus group 9)

Several adolescents recounted experiences of self-harm to alleviate overwhelming emotional pain, both direct and indirect. One participant talked about how they exercised in excess to minimize anxiety, for example: …then I exercised until I almost fainted, to get rid of it [the pain] … I felt very bad at times. It was not the best way to solve it (Girl, Focus group 2). Others spoke about self-harm through cutting. Although there was also an awareness that self-harming only provided short-term relief and was not a positive solution:

It sounds silly to others, but when I felt so bad, I felt so bad that I cut myself. And then it felt good in the moment. But it didn’t feel good after … when you realized what you had done, so it was kind of both. (Girl, Focus group 4)

### “Bite the Bullet” and Endure the Pain

The adolescents also said that they dealt with pain by “biting the bullet”—enduring the pain. They felt that their experiences of pain, including infidelity and physical exercise, ultimately made them stronger.

There was a certain pride in these stories, how they had endured, for example, muscle fatigue: “But muscle fatigue and such, you still feel if you’re in pain, you just ‘but then I’ve done something’. It still feels very good afterwards” (Girl, Focus group 3); infidelity: “I’ve been through infidelity, and then [I] had to break up because I don’t think you should accept it, and then it hurts, but it was a good pain because I became so much stronger” (Girl, Focus group 2); or overcoming anxiety attacks, for example:

I think when you’re in the middle of an anxiety attack, it hurts in different places in the body … then you just have techniques on how to deal with it and then when it has passed, you have overcome it and feel that you are stronger than the anxiety. (Girl, Focus group 5)

By enduring pain, they could overcome both the pain itself and fear of the pain. The pain in the moment may not feel good, but it can be viewed as a learning experience that can benefit a person in the long run:

I think that maybe the pain is not good [in] the moment, but I think that this kind of mental pain can also strengthen you. You have with you everything you have been through. In the future, you might still feel that you are somehow still happy that it happened or something like that, because you have still learned something. (Girl, Focus group 1)

### Use Painkillers and Complementary Treatment

Painkillers, analgesics, and anti-depressives were accepted as possible means to alleviate pain. However, efficacy and the possibility of addiction were considered. Many adolescents discussed alternative and complementary methods to alleviate pain.

The adolescents described use of alternative relief methods as well as painkillers—especially paracetamol and ibuprofen—to manage their pain, for example, for period pain: “I usually take paracetamol when I have pain, but if I have, for example, period pains, I usually take a heat pad and put it on or massage where it hurts, yes” (Girl, Focus group 5). Some adolescents included anti-depressive drugs in discussion, illustrating the frequent cognitive and physical crossover between stress and pain. But these did not always help:

Yes, I think I felt bad mentally, so I went on sertraline. They thought it would help me, but it didn’t help at all. It only got worse… I had pain inside my body, so they explained to me. They thought it would help. So, I went on it for a while but then it only got worse—So that I had to stop taking them. And then it got better when I stopped taking medicine. (Girl, Focus group 4)

Others believed that it was best to avoid drugs as much as possible. Some urged caution when using painkillers, linking this with the risk of becoming addicted or experiencing side effects, and most adolescents had a positive attitude toward trying alternative treatments, such as meditation, massage, heat pads, and increased water intake:

One adolescent, who was managing chronic pain in their life, relayed that they felt they were addicted to painkillers and were trying to be careful not to aggravate the addiction:

Uh, well, I kind of live my life on painkillers. Because I wake up with back and knee pain every day… if it works, it works. I’d rather try to find more natural ways than to turn directly to medicines. I have addiction in the family to narcotics. It's easier for me to be able to become addicted. And that's why I have, I feel that there is something more… As natural and neutral as you said, you can drink water for a headache, do that instead of taking ipren [Ibuprofen] and alvedon [Paracetamol]. If you have a cold, yes, but get a heat pad, eh, or get like icepacks, or something like that. (Boy, Focus group 7)

### Ask for Help from Others

The adolescents said that talking to someone about their pain was helpful. Although some did not feel comfortable sharing their life with others, they believed it necessary to talk about the pain, recognizing that it can be a great help to receive support from someone, and is a significant part of the healing process.

Many of the young people spontaneously volunteered their mother as the person they primarily turned to, but also mentioned friends, other family members, or professionals:

Manage the pain by talking to your parents… they have also been our age so they might be able to give tips on what they have done. If you get a lot of anxiety and all such problems, you can seek help yourself; for example, from a counselor or a psychologist. (Girl, Focus group 6)

The professionals mentioned included school nurses, school counselors, doctors, and psychologists. Talking to someone about a pain experience could result in adopting a new perspective on a problem and strategizing and implementing behavioral alterations to avoid future pain:

I think it is absolutely necessary to talk out, to deal with pain. Not everyone gets fixed by talking it out, but I think it can be a big help along the way, talking it out with someone. It could be with a friend. I think it means a lot to get just a little support, that it is a huge part of the road. (Boy, Focus group 9)

## Discussion

The findings of this focus group study with adolescents aged 16–19 illustrate pain experiences that encompass both the physical and emotional, alongside descriptions of a range of ways to mitigate pain in their lives.

In the study by Persson et al. (2021) where experience of pain was explored with 10–12-year-olds, similar themes were identified as with the adolescents: physical and emotional pain, pain related to schoolwork, and the benefits of pain. However, the overlap of emotional and physical pain is more apparent with the older group, and the range of relationships from which the pain of grief or conflict could arise is wider. In addition, critical strategies to manage pain are far more apparent in the adolescent narratives.

For the adolescents, physical pain was mainly associated with the increasing academic workload in their lives—staying in one position for too long as they study—or the stress of assignments and deadlines leading to headaches and stomachaches. The school environment and schoolwork are perhaps unsurprisingly evident in the narratives of both age groups. However, there are developmental differences. The younger children speak of the stressful environment, whereas the adolescents speak more of the demands of increasing academic demands as they approach significant academic milestones.

The younger children (Persson et al., 2021) described conflict with peers, and although there were examples in the older group of pain through being bullied, peer conflict was less apparent with the older group—more often they described situations where they would help others, either as a distraction from personal pain, or stepping into a situation to help another that incurred injury. This shift is in-keeping with a growing sense of values and sense of place in society, and receding egocentrism. In addition, romantic relationships were almost absent from the young groups’ narratives, and very much present in the older group. The pain of loss of loved ones, apparent in both, was more philosophically considered by the older group—who recognized grief as part of being “human.” Difficult childhoods were hinted at by some of the adolescents, which was indicative of consideration of what has shaped their lives thus far, and how to manage this going forward.

These differences are reflective of Erikson and Erikson's (1998) stages of psycho-social human development. Each of Erikson and Erikson's eight stages identified across the lifespan is influenced by biological, psychological, and social factors. The younger age group is in stage four, industry versus inferiority, where the peer group gains significance and is a major source of the child's self-esteem. This is evident in the younger age group's source of emotional pain, mostly being located in peer conflict. Stage five, identity versus role confusion, is typical for the teenage years. During this stage, the young person explores personal goals, values, and identity. In the adolescent group altruistic acts, learning what they do and do not want from interpersonal relationships, and integrating learning through loss were increasingly apparent.

Both age groups were able to express that while pain was a negative feeling, it could be positive: alerting to actual or potential injury or learning about relationships through conflict with family members or friends; and with the older group, romantic relationships. While both age groups spoke about the loss of loved ones, the older age group explored more philosophical aspects, such as loss being a natural part of being human, and the value of positive memories. This emotional intelligence and broader understanding of pain indicates that these young people are also entering into Erikson and Erikson's sixth stage, intimacy versus isolation, where people develop sustained, fulfilling relationships with people who are not family—including friends and adults, as well as romantic relationships. These expressions of emotional pain spoke to a growing life experience, where learning through experience could be applied to life going forward (Bekaert & Appleton, 2021).

In discussions around managing pain, cognitive development was also apparent in comparing the two age groups. While the younger age group cited various modes of pain relief—these remained “concrete” (Piaget, 1969)—focusing on what is observable in the physical world, that is, medicine stops pain. Commensurate with their further life experience and social interaction the adolescents described a range of methods to alleviate pain—medication, alternative modes, even self-harm. In addition, their ability to think hypothetically, and therefore, critically, facilitated consideration of avoiding medication addiction, and the short-term relief of self-harm. Crucially, the adolescents also spoke of a range of sources of help, widening out from family. These included professionals including the school nurse. This is an encouraging finding as other studies have suggested that teens generally do not like to seek help, either through not recognizing there is an issue, or the desire for independence overrides a desire for support (Bekaert & Appleton, 2020; Bekaert & SmithBattle, 2016).

### Implications for School Nursing and Future Research

The findings from this study emphasizes the importance of a biopsychosocial understanding of pain, which considers the interplay between biological, psychological, and social factors (Nicholas, 2022). How the adolescents expressed their experience of pain, and how they dealt with it, also highlights important developmental aspects that can inform school nurse practice with this specific age group. School nurses can offer a range of practical and emotional support, as well as identify when further specialist support is needed. This can be through group health promotion, for example, health education can be offered to help adolescents manage their mental and physical health as academic work increases. Such as through practical advice regarding regular breaks, hydration and exercise. As romantic relationships become relevant, health education regarding what is a healthy relationship, respect, and consent can be offered. The school nurse is available for one-to-one support. Teens value a safe space and trusted adults with whom to explore health-related issues and with the school nurse. School nurses’ scientific knowledge and therapeutic ability are ideally placed to offer this support (Pavelová et al., 2021). The mental health needs of teenagers are a significant current concern both globally and nationally, particularly in the wake of the Covid-19 pandemic and associated impacts on young people mental health (Chen et al., 2022). School nurses play an important role in the provision of mental health services in the school environment (Bohnenkamp et al., 2015). For students who are experiencing, or have experienced grief, school nurses are part of an interprofessional school support team (Edwards et al., 2023). For students who disclose difficult childhoods and are beginning to voice this pain in their lives, the school nurse can offer a safe space to explore these experiences, and to refer to specialist support as needed, promoting positive health behaviors and decreasing the negative side effects of trauma (Sypniewski, 2016).

Promoting positive health behavior is a key public health role of the school nurse. The school nurse can facilitate both pain prevention and relief. This age group demonstrated knowledge of a variety of modes of pain relief and critical thought regarding constructive versus destructive modes, that is, self-harm. The school nurse can take a mentorship approach toward facilitating adolescents’ understanding of positive pain-mitigating strategies as opposed to a directive approach that might be suitable with a younger age group. Working alongside the adolescents to explore positive strategies and steer away from negative ones can be a more successful motivational approach (Beckwith & Beckwith, 2020). A study by Karman et al. (2015) found that school nurses felt ill-equipped to support young people with self-harm, and that training was needed in this specific area.

The school nurse work as a positive resource with the flexibility and growth of critical thought across adolescence (Piaget, 1968) and the plasticity of the teenage brain (Sercombe, 2010). Working alongside adolescents, school nurses can facilitate positive pathways to deal effectively with the expressed causes of pain in their lives. Support that recognizes a young person's growing independence, and that equips them with learning and positive strategies offers early intervention to promote well-being and positive strategies to face life's ongoing challenges.

## Strengths and Limitations

The focus group approach gave the opportunity to hear the experiences of adolescents and for the young people to develop discussion around a group dynamic. One-to-one interviews may have offered the opportunity to explore more personal, deeper aspects of pain, for example, when young people expressed childhood pain, it was not ethically possible to explore such comments in a group setting due to the sensitive subject nature.

The focus groups took place in two schools, and participants self-selected to participate. Therefore, findings may not be transferable to the general adolescent population. However, the results offered a range of experiences of pain, and strategies to manage pain which may be useful knowledge when school nurses encountering adolescents. Focus groups explored both stress and pain within a short time frame which may have compromised full exploration of the concepts. We were bound by the times offered by the school, nevertheless, rich data was achieved in both areas to begin to explore the adolescents’ experience of stress and pain.

Five of the focus groups were mixed, and four were females only. The lack of male-only groups and the fact that there only were 9 males and 32 females who participated must be taken into account. Aspects of male voices may be missing.

The interview guide was developed for use with children and adolescents aged 10–19. Using the same interview schedule across the age range could be a limitation as it might not include developmental nuance. However, this is equally a strength as the guide is validated through previous use and gives scope for comparison between the age groups.

The qualitative content analysis with Hsieh and Shannon (2005) is beneficial to use when the data is broad, and the goal is to identify similarities and differences in the material. The analysis was conducted initially with two researchers, and then jointly with all four researchers until consensus was reached. This collaborative approach strengthens the analysis.

## Conclusion

The adolescents in this study described how pain expressed itself in their lives and their ways of dealing with pain. They described pain as a warning-bell, as related to school and work or interpersonal relationships, as well as a sign of humanity. They dealt with pain by turning attention elsewhere, enduring the pain, using painkillers and complementary treatments and also asking for help from others.

This study has specifically focused on adolescents’ experience of pain in their evolving cognition and life experience. The findings from this study serve to inform school nursing practice with this age group in relation to managing pain. School nurses have a knowledge of child development that recognizes that specific health-related areas will become salient at different developmental stages and that different support strategies are required for different age groups.

## Appendix

**Interview guide**

- What do you do to feel good?
- What do you think of when you hear the word pain?
- Can you describe situations when pain has been good?
- Can you describe situations when pain has been difficult?
- What can be done to manage pain? Can you give examples?
- How do you reason about using painkillers?
- Is there anything else related to well-being, pain and medication use that you think would be important to discuss?
